# Cancer stem cell phenotype relates to radio-chemotherapy outcome in locally advanced squamous cell head–neck cancer

**DOI:** 10.1038/bjc.2012.33

**Published:** 2012-02-14

**Authors:** M I Koukourakis, A Giatromanolaki, V Tsakmaki, V Danielidis, E Sivridis

**Affiliations:** 1 Department of Radiotherapy/Oncology, Democritus University of Thrace and University General Hospital of Alexandroupolis, PO Box 12, Alexandroupolis 68100, Greece; 2 Department of Pathology, Democritus University of Thrace and University General Hospital of Alexandroupolis, Alexandroupolis, Greece; 3 Department of ENT, Democritus University of Thrace and University General Hospital of Alexandroupolis, Alexandroupolis, Greece

**Keywords:** CD44, CD24, Oct4, integrin-*β*1, ALDH1, head–neck cancer radiotherapy

## Abstract

**Background:**

Cancer stem cells (CSCs) tend to repopulate malignant tumours during radiotherapy and, therefore, prolongation of the overall treatment time may result in radiotherapy failure. Thus, an estimate of the number of CSCs in tumour biopsies may prove most useful in predicting resistance to radiotherapy and a guide for development therapies aimed to eradicate a cancer cell population with effects on radiotherapy-related cancer regrowth.

**Methods:**

The CSC population was investigated semi-quantitatively in 74 locally advanced squamous cell head–neck cancers (HNSCC) from an equal number of patients, treated with accelerated platinum-based radiotherapy. A standard immunohistochemical technique and the CSC markers CD44, CD24, Oct4, integrin-*β*1 and aldehyde dehydrogenase isoform 1A1 (ALDHA1) was used, in parallel with the proliferation marker MIB-1. The results were correlated with the site of the tumour, the MIB-1 index, the tumour grade and stage, and prognosis.

**Results:**

The expression of CD44, CD24 and Oct4 were significantly associated with the MIB-1 proliferation index. In addition, the CD44 was linked with the better differentiated HNSCC. The CD44, Oct4 and integrin-*β*1 were all associated with poor prognosis but, in a multivariate analysis, the integrin-*β*1 had an independent statistical significance in terms of local relapse, distant metastases and overall survival. Interestingly, ALDH1 was associated with favourable prognosis.

**Conclusion:**

CSC markers are linked with poor radiotherapy outcome in HNSCC, with integrin-*β*1 being the strongest and independent prognostic factor. Targeting CSC molecules with monoclonal antibodies or pharmaceutical agents may prove important for the treatment of HNSCC.

Like normal tissues, malignant epithelia contain a subpopulation of cells with regenerative abilities under cytotoxic stress. Such cancer stem or clonogenic cells tend to repopulate tumours during the course of radiotherapy and, thus, by extending a standard radiotherapy scheme over a period of 7 weeks may become a major cause of radiotherapy failure (Trott, 1990). It is postulated that quantification of cancer stem cells (CSCs) could be useful in identifying subgroups of patients that would benefit from accelerated radiotherapy schemes or from biological interference targeting this very cancer cell population. This phenomenon may also have important implications in the chemotherapy efficacy, as CSC repopulation may occur between chemotherapy cycles (Davis and Tannock, 2000).

The CD44 is a cell adhesion glycoprotein functioning as a transmembrane receptor for the extracellular matrix component hyaluronan. It participates in epithelial cell–stroma interactions that are important in tumour invasion and metastasis (Bourguignon 2008; Naor *et al*, 2008). Current experimental evidence suggests that CD44 expression characterises a subset of cancer cells with stem-cell-like properties. This has been confirmed in several human tumours (Krishnamurthy *et al*, 2010; Xu *et al*, 2010), including squamous cell head–neck cancers (HNSCC) (Krishnamurthy *et al*, 2010). CD24, on the other hand, is a highly glycosylated cell adhesion molecule (Hough *et al*, 1994) that binds to P-selectin and is involved in tumour cell invasion and metastasis (Aigner *et al*, 1997; Lim, 2005). The CD44+/CD24− phenotype is believed to characterise the triple-negative breast carcinoma and is linked with poor prognosis (Zhou *et al*, 2010), which was also confirmed in a recent study from our group (Giatromanolaki *et al*, 2011).

Aldehyde dehydrogenase isoform 1A1 (ALDH1A1), an enzyme oxidising aliphatic aldehydes, has been also recognised as a universal marker of CSCs (Marcato *et al*, 2011) and has become target of therapeutic intervention (Visus *et al*, 2011). OCT4 (Pou5f1) is a transcription factor highly expressed in undifferentiated embryonic stem cells (Niwa *et al*, 2000) that maintains pluripotency in collaboration with other transcription factors such as Sox2 and Nanog (Boyer *et al*, 2005). Integrin-*β*_1_ (CD29) is a transmembrane glycoprotein that following binding to the *α*_1−6_ subunits forms functional receptors binding to the extracellular matrix. This is an important molecule involved in haemopoietic stem cell cycling (Zhang *et al*, 2003), being recently reported also to characterise CSCs (Maitland and Collins, 2008).

In this study, we analysed the expression patterns of multiple antigens characterising CSCs, in a series of patients with locally advanced HNSCC treated with accelerated platinum-based radio-chemotherapy, in an attempt to evaluate the role of such a cancer population in the efficacy of radiotherapy and the metastatic ability of these tumours. The proliferation marker MIB-1 was also assessed to test whether tumours with high percentage of stem cells bear an increased growth fraction but also to assess any effect of this feature on the radiotherapy outcome.

## Materials and methods

A total of 74 biopsy specimens of HNSCC from an equal number of patients were recruited in this analysis. The material was retrieved form the archives of the Department of Pathology, Democritus University of Thrace and University Hospital of Alexandroupolis, Alexandroupolis, Greece. All biopsies had been fixed in 10% formal saline and processed routinely to paraffin wax. The original haematoxylin and eosin-stained sections were reviewed and the cases selected were confirmed as being squamous cell carcinomas. In all, 68 patients were male (91.9%). The median age ranged from 42 to 86 years (median 68). All patients had inoperable or recurrent (after surgery) disease, and were treated with platinum based (cisplatin 35 mg m^−2^ per week or oxaliplatin 60 mg m^−2^ per week) hyporfractionated accelerated conformal radiotherapy supported with daily amifostine (HypoARC) using a concomitant boost technique (2.7 Gy × 20–22 fractions, within 4–5 weeks). In all, 15 out of these cases also received cetuximab at the approved dosage of 250 mg m^−2^ per week). Appropriate shielding of the spinal cord was considered in the planning to keep the biological dose to 44 Gy. Detailed analysis of the radiation schedule and its radiobiological relevance has been reported previously (Koukourakis *et al*, 2010). The median follow-up period of the patients was 24 months (4–80 months). Written informed consent was obtained from the patients, and the Institutional Ethics and Scientific Committee approved the study.

The small number of patients does not allow a separate analysis to assess whether the addition of cetuximab to platinum affects the overall relation of stem cell markers with radiotherapy outcome. Nevertheless, there was no statistically significant difference in response rates or local relapse survival rates between patients receiving or non-receiving cetuximab.

Response to treatment of measurable lesions was assessed with CT scan 2 and 4 months after treatment completion. Complete disappearance of the local and regional disease or remnant scar tissue measuring <5% of the initial dimensions was considered as complete response. All other cases were characterised as an incomplete response and underwent further chemotherapy. Patients entered a regular follow-up with CT scan of the splanchinc cranium, neck, chest and upper abdomen performed every 6 months.

### Immunohistochemistry

For the detection of CD44 antigen the mouse monoclonal antibody Ab6124 (Abcam, Cambridge, UK) was used, at a dilution of 1 : 100. Detection of the CD24 antigen was achieved using the mouse monoclonal CM323 antibody (Biocare Medical, Concord, CA, USA) at a dilution of 1 : 100, while that of the proliferation marker Ki67 the anti-Ki67 monoclonal antibody (clone MIB-1, YLEM, Rome, Italy) was applied at a dilution of 1 : 75. For the ALDH1 protein detection we used the EP1933Y (ab52492, Abcam) rabbit monoclonal antibody, raised against a synthetic peptide corresponding to residues near the C terminus of human ALDH1A1 protein, at a dilution of 1 : 200. For the embryonic stem cell marker Oct4, we used the rabbit polyclonal ab19857 (Abcam) raised against a synthetic peptide derived from residues at the C terminus of the human Oct4, at a dilution of 1 : 200. The mouse monoclonal 4B7R antibody (ab3167, Abcam), at a dilution of 1 : 40 was used to detect the integrin-*β*_1_.

Sections were cut at 3 *μ*m and stained as follows: they were dewaxed and rehydrated in graded alcohol solutions. For heat-induced epitope retrieval, the sections were placed in citrate buffer (1 : 10 dilution, pH 7.2) and heated at 120 °C for 3 × 5 min. Endogenous peroxidase activity was neutralised using Peroxidase Block for 5 min. The non-specific binding was blocked by pre-incubation with Protein Block for 5 min at room temperature (Novocastra Laboratories Ltd, Newcastle upon Tyne, UK). Slides were then incubated overnight at 4 °C with the primary antibody. The slides were washed with PBS (2 × 5 min) and then incubated with Post Primary Block (that enhances penetration of the subsequent polymer reagent) for 30 min at room temperature (Novocastra Laboratories). Washed with PBS for 2 × 5 min and incubated with NovoLink polymer for 30 min at room temperature (Novocastra Laboratories). This recognises mouse and rabbit immunoglobulins and detects any tissue-bound primary antibody. After extensive washing with PBS (2 × 5 min), the colour reaction was developed in 3,3′-diaminobenzidine for 5 min. The sections were then counterstained with haematoxylin, dehydrated and mounted. Normal (species dependant) immunoglobulin G was substituted for the primary antibody as negative control. Staining with omission of the primary antibody was also performed as negative control.

### Assessment of staining

Evaluation of the CD44, CD24 and integrin-*β*1 expression was based exclusively on membrane staining, although cytoplasmic reactivity was also noted. Evaluation of ALDH1 and Oct4 was based on their cytoplasmic expression. The percentage of cancer cells with membranous staining was estimated semi-quantitatively by two observers (AG and ES) in all optical fields at × 200 magnification and the mean value was used to score each case. Observers were blinded to the clinical data, and discrepancies between observers were resolved on the conference microscope. Cases with a score higher (and/or equal) to the median value were considered as bearing high percentage of positive cells so that two groups the low *vs* the high reactivity were considered for analysis.

For the MIB1 staining, the percentage of cancer cell nuclei stained with the antibody was assessed in all × 200 optical fields and the mean value was used to score each case. Cases with a score ⩾median value were considered as having a high proliferation index.

### Statistical analysis

Statistical analysis and graphs were performed using the GraphPad Prism 5.0 (San Diego, CA, USA) and the SPSS 14.0 packages (SPSS Inc., Chicago, IL, USA). Survival curves were plotted using the method of Kaplan and Meier, and the log-rank test was used to determine statistical differences between life tables. A Fisher's exact test and the unpaired two-tailed *t*-test were used for testing relationships between categorical tumour variables, as appropriate. Multivariate analysis was applied to assess the independent predictive significance of variables on response to therapy. A Cox proportional hazard model was used to assess the effect of assessed parameters on local relapse and overall survival. A *P*-value of <0.05 was used for significance.

## Results

### Patterns of expression

**Figure 1:**
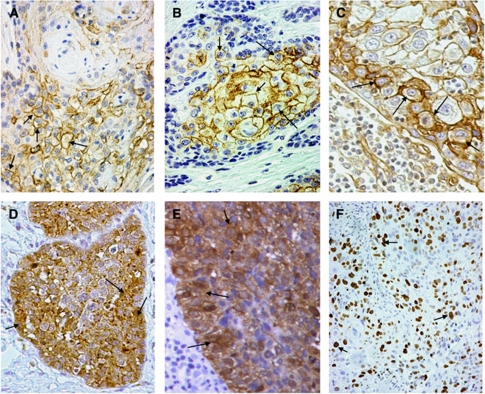
Immunohistochemical images of HNSCC with focal expression of stem cell markers and MIB1 (arrows): membrane staining using the anti-CD44 antibody (**A**), membrane staining using anti-CD24 antibody (**B**), membrane staining using anti-integrin-*β*1 antibody (**C**), cytoplasmic staining using the anti-Oct4 antobody (**D**), cytoplasmic staining using the anti-ALDH1 antibody (**E**) and nuclear staining using the MIB1 antibody (**F**).

The expression of CD44, CD24 and integrin-*β*1 antigens was both membranous and cytoplasmic in squamous cancer cells (Figures 1A–C), although only the membranous pattern was taken into account for statistical analysis. The percentage of cancer cells with membranous CD44 expression ranged from 0 to 100% (median 40, mean±s.d. 44±35), with 46/74 (62.2%) cases expressing CD44 in ⩾40% of cancer cells and thus considered as being positive. The percentage of cancer cells with membrane CD24 expression ranged from 0 to 50% (median 0, mean±s.d. 3±8), with 15/139 (20.2%) cases being positive, that is, expressing CD24 in ⩾10% of cancer cells. The proportion of cancer cells with membrane integrin-*β*1 expression ranged from 0 to 80% with a median value of 5% (mean 13.4±19). Cases with a score >5% (all were >20%), thus 26/74 (35%) were considered as positive for integrin-*β*1.

The expression of Oct4 and of ALDH1 was cytoplasmic (Figures 1D and E), for Oct4 the median percentage of cancer cells expressing the antigen was 20% (range 0–100%, mean 33.5±35). Using this cutoff point (>20%), 31/74 cases had high Oct4 reactivity. Aldehyde dehydrogenase isoform 1A1 was expressed in 0–90% of cancer cells (median 0%, mean 12.7±22). Out of 74 cases, 31 (41.9%) expressed ALDH1 in >5% of cancer cells and these cases were considered as positive.

The percentage of cancer cells with nuclear MIB-1 expression (Figure 1F), ranged from 0 to 90% (median 10%). In all, 41 cases (55.4%) showed nuclear positivity in ⩾10% of cancer cells and these were considered to have high proliferation index.

For all parameters assessed, there was a good correlation of the scores given by the two observers (correlation analysis: *P*<0.0001, *r*>0.89). Table 1 shows the analysis of tumour samples according to the percentage of cancer cells expressing the studied stem cell markers.

### Association among stem cell markers and MIB1

In-group analysis (Table 2), using the cutoff point mentioned in the methods, OCT4 was directly related to integrin-*β*1 expression (*P*=0.05). A trend was noted for CD44 to be directly related with CD24, Oct4 and ALDH1 (*P*>0.09).

High proliferation MIB1 index was significantly related to CD44 (*P*=0.03), CD24 (*P*=0.04) and Oct4 (*P*=0.02) expression. In a multivariate model of all stem cell markers against MIB1 proliferation index, CD24 and integrin-*β*1 were significantly and independently linked with high index (*P*=0.05, *t*-ratio 1.96 and *P*=0.03, *t*-ratio 2.12, respectively).

### Association with histopathological variables

Table 3 shows the association of stem cell markers with primary tumour location, T and N-stage and histological grade. CD44 expression was linked with low-grade tumours (*P*=0.02), while there was a trend for CD24 to associate with advanced T-stage (*P*=0.08). Of interest, all five recurrent tumours overexpressed CD44 (*P*=0.14), and Oct4 (*P*=0.01) and 4/5 overexpressed integrin-*β*1 (*P*=0.04). There was no association with primary tumour location.

### Response analysis

Two months following the radio-chemotherapy, complete response of the local and/or nodal disease was obtained in 60/74 (81.1%) cases. Analysis of stem cell markers and other histopathological variables according to CR is shown in Table 4. High presence of CD44+ cells and positive node disease were significantly linked with incomplete response after therapy (*P*=0.04). In a bivariate model, both parameters maintained their independent predictive relevance (*P*=0.02 and *P*=0.02, respectively). There was a trend for Oct4 and integrin-*β*1 to link with incomplete response (*P*>0.06).

### Survival analysis

Table 5 shows the univariate and multivariate analysis of local progression-free interval, metastasis-free interval and overall disease-specific survival. Univariate analysis refers to Kaplan–Meier analysis values. Multivariate analysis comprises only the parameters that had significant impact at univariate analysis. Hazard ratios refer to comparison of high score *vs* low.

**Figure 2:**
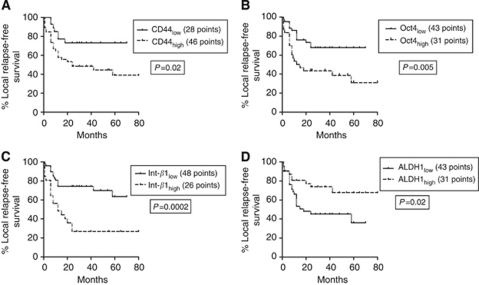
Kaplan–Meier LRFS curves according to CD44 (**A**), Oct4 (**B**), integrin-*β*1 (**C**) and ALDH1 expression (**D**).

**Figure 3:**
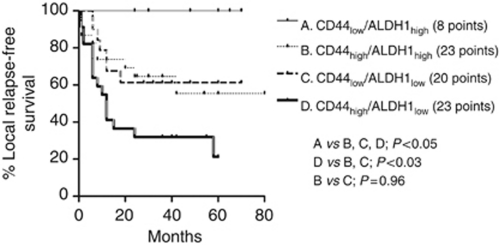
Kaplan–Meier LRFS curves with double stratification according to CD44 and ALDH1 expression.

At univariate analysis of local relapse-free survival (LRFS), high integrin-*β*1 (*P*=0.0002), Oct4 (*P*=0.005) and CD44 (*P*=0.02), as well as low expression of ALDH1 (*P*=0.02), were linked with significantly worse prognosis (Figure 2). Advanced T3, 4 stage was also linked with local relapse (*P*=0.02). In multivariate analysis, integrin-*β*1 and ALDH1 expression, as well as T-stage maintained an independent prognostic significance. Double stratification analysis of LRFS according to CD44 and ALDH1 was also performed and is shown in Figure 3. Tumours with high CD44 and low ALDH1 expression had the poorest LRFS, whereas ALDH1 overexpressing tumours with lack of CD44 expression had the best LRFS.

Univariate analysis of the distant metastasis-free survival showed that N-stage, Oct4 and integrin-*β*1 expression were significantly linked with distant metastasis. In multivariate analysis only integrin-*β*1 expression maintained independent prognostic relevance (*P*=0.01, risk ratio 5.72).

Univariate analysis of disease-specific overall survival showed a significant association of integri *β*1 high expression with poor outcome (*P*=0.01, risk ratio 2.56). T-stage and oct4 and ALDH1 expression showed a trend (*P*>0.06). Multivariate analysis was not feasible as the only significant value at univariate was that of integrin-*β*1.

## Discussion

Cancer stem cells (CSCs) are generally thought to be the source upon which tumour survival and re-growth relies, following the depopulation induced by radiation or cytotoxic agents (Davis and Tannock, 2000; Milas and Hittelman, 2009). In a subset of tumours, at least, the reduction of the overall treatment time by 2–4 weeks results in improved radiotherapy efficacy, as shown in randomised trials in HNSCC (Dische *et al*, 1997; Fu *et al*, 2000). As accelerated tumour repopulation is a major cause of radiotherapy failure, especially when prolonged radiotherapy schedules are applied, it may be suggested that tumours containing a large compartment of CSCs that can enter a rapid proliferation phase during the radiotherapy course could benefit from short radiotherapy schedules (Geara *et al*, 1991). Nevertheless, the inherent ability of stem cells to respond by entering active cell cycle or even the high intrinsic radioresistance of such cells may abrogate the effect of acceleration regardless the extent of their presence in the tumour. Indeed, such clonogenic cells rapidly repopulate cancer cell cultures in serial irradiation/re-growth assays (Zhang *et al*, 2008). Although such assumption is based on a strong clinical rational, the identity of these stem cells and the nature of their interaction with radiation remains obscure.

During the past decade a large number of cell antigens that characterise CSCs have been identified (Gil *et al*, 2008). A large field of translational research has, therefore, been opened aiming to the characterisation of such clonogenic cells in human malignancies and their subsequent targeting for therapy. Among these antigens, CD44 and ALDH have focused the attention in HNSCC (Prince *et al*, 2007). In a study by Lo *et al* (2011), CD44+ cells displayed CSC-like properties in HNSCC, also exhibiting higher radio-resistance. The tumourigenicity of CD44+ cells of HNSCC seems also to increase when such cells co-express additional markers such as the c-met or the ALDH (Krishnamurthy *et al*, 2010; Sun and Wang, 2011). An increased metastatic potential of CD44+ HNSCC cells has been also reported in an *in vivo* experimental model, although the invasive ability of such cells did not seem to increase (Davis *et al*, 2010).

In this study, an extensive presence of CD44+ cancer cells in locally advanced HNSCC was directly linked with increased proliferation index. The same finding was also applied for an additional marker of CSCs, namely the Oct4 and CD24. This latter has been recently linked with poor survival in squamous cell cervical cancer (Kwon *et al*, 2007; Sung *et al*, 2010). CD24 expression also showed a marginal association with larger tumours. The close association of CD44, CD24 and Oct4 with cancer cell proliferation highly supports the assumption that these are indeed markers of clonogenic cells.

A rather unexpected finding was that CD44 positivity was significantly linked with well-differentiated tumours. This may show that CD44+ cells may represent a CSC population characterising mostly the well-differentiated HNSCC. Interestingly, the Danish Head Neck Cancer Study Group randomised trial showed that mild acceleration of radiotherapy was more effective in well-differentiated HNSCC cases, expected to have a higher percentage of CD44+ cells according to our study (Eriksen *et al*, 2005). Whether distinct phenotypic imprint characterises stem cells that may give growth to well or poorly differentiated HNSCC and whether these have distinct dose fraction and time-dependent radio sensitivities is a hypothesis that needs further investigation.

Another interesting finding was that recurrent tumours had a significantly higher percentage of cancer cells expressing two stem cell markers, namely the Oct4 and integrin-*β*1. CD44 was also prevalent in these tumours, which is in accordance with a study by Joshua *et al* (2011) who found an increased frequency of CD44+ cells in recurrent HNSCC. This stresses the importance of cells with this phenotype to provide the seed for subsequent tumour re-growth after complete surgery. Moreover, tumours with intense presence of Oct4 and integrin-*β*1 had a significantly higher incidence of distant metastasis, showing that, apart from their clonogenicity, such cells have important migratory capacity. Eradication of such a sub-population emerges, therefore, as an important quest for the postoperative or post-radiotherapy period. Targeting integrins with monoclonal antibodies or pharmaceutical agents, such as vinculin activators, may prove of importance for the treatment of such tumours (Nelson *et al*, 2011; Schmid *et al*, 2011).

Analysis of the efficacy of radio-chemotherapy showed that an extensive expression of stem cell markers, that is, integrin-*β*1, Oct4 and CD44+, by cancer cell was linked with significantly poorer local progression-free interval. This finding is in accordance with previous studies. In a study by De Jong *et al* (2010), CD44 was the only biological factor that significantly correlated with response to radiotherapy in early stage laryngeal cancer. Whether the above observation of stem cell marker association with reduced radiotherapy efficacy is a result of increased clonogenic repopulation or of an enhanced intrinsic radioresistance of these cells is unknown. The fact that despite the accelerated radiotherapy regimen applied in this study, CD44 remained a predictor of local relapse, suggests that reduced radiosensitvity may characterise this tumour sub-population. Indeed, CD44+/ALDH+ cells isolated from HNSCC exhibit increased radioresistance and reversal of this phenotype by a STAT3 signalling blocker restored radiosensitivity of cancer cells (Chen *et al*, 2011). The unexpected finding that ALDH1 expression was linked with better radiotherapy outcome suggests that not all stem cell markers characterise the same cell population and that not all stem cells are resistant to radiation. Indeed, Zielske *et al* (2011) found that in different breast tumours ALDH1-positive CSCs exhibit an individual radioresistance, the radiotherapy being able to easily eradicate CSCs in some tumours but being incapable to do that in others. It may be that clonogenicity varies among cancer cells bearing distinct stem cell markers and that so does their sensitivity to altered fractionation. Indeed, there was no association among the different markers used, with the exception of Oct4 and integrin-*β*1. It may be that more than one sub-populations with stem cell abilities may exist in the same tumour. Overall, the most potent stem cell marker in this series of squamous cell carcinomas that affected both local control and survival, independently of all histopathological variables, was integrin-*β*1.

An important finding that emerges from this immunohistochemical study is the extensive expression of the putative stem cell markers applied in some tumours (Table 1). This questions the validity of the used markers to identify exclusively the stem cells in a tumour. It may be that in some tumours terminally differentiated cancer cells can preserve the expression of the stem cell markers. Such a hypothesis could provide a rational for the association of ALDH with good prognosis or of CD44 with well-differentiated neoplasms. Another explanation is that stem cell markers are also proteins that may have a defined role in cell metabolism (like ALDH) or cell migration (such as integrins and hyaluronan receptors), the stimulated expression of which may occur also in differentiated cancer cells under hypoxic or acidic conditions that prevail in growing tumours. It is, therefore, possible that although stem cell markers characterise clonogenic cells, their overexpression may also occur as a stress response in differentiated cells. Moreover, as such proteins may have additional biological roles in the survival, growth and migration of tumours, the associations found with radiotherapy outcome may be a result of radioresistance conferred by this very biological process and not a result of a repopulation overactivity of stem cells identified by these markers.

Despite the heterogeneity of HNSCC included in the series of patients herein analysed, which is certainly a limitation of the study, our findings suggest that integrin-*β*1 and Oct4 are stem cell markers for HNSCC linked with resistance to radiotherapy. CD44 when overexpressed in HNSCC characterises tumours with good differentiation, increased proliferation rate and resistance to accelerated radiotherapy. Whether targeted therapies aiming to the elimination of such tumour subpopulation or STAT3 inhibitors shown to induce differentiation of immature cell phenotype to a more radiosensitive cell population can improve the efficacy of radiotherapy is a hypothesis supported by the current findings.

## Figures and Tables

**Table 1:** Distribution of tumour samples according to the percentage of cancer cells expressing the stem cell markers

	**Number of cancer samples (%)**			
	**Percentage of cancer cells expressing the stem cell marker**			
	**0%**	**5–10%**	**20–40%**	**⩾50%**
*Marker*				
CD44	17 (23)	8 (11)	13 (18)	36 (48)
CD24	59 (80)	12 (16)	2 (3)	1 (1)
Oct4	25 (34)	8 (11)	14 (19)	27 (36)
Integrin-*β*1	32 (43)	16 (22)	20 (27)	6 (8)
ALDH1	42 (57)	13 (18)	10 (13)	9 (12)

Abbreviation: ALDH1=aldehyde dehydrogenase isoform 1A1.

**Table 2:** Association among stem cell markers and MIB1 proliferation index

	**CD44**			**CD24**			**Oct4**			**Integrin-** *β* **1**			**ALDH1**		
	**Low**	**High**	***P*-value**	**Low**	**High**	***P*-value**	**Low**	**High**	***P*-value**	**Low**	**High**	***P*-value**	**Low**	**High**	***P*-value**
*CD24*															
Low	25	34	0.14												
High	3	12													
															
*Oct4*															
Low	20	23	0.09	35	8	0.77									
High	8	23		24	7										
															
*Integrin-β1*															
Low	21	27	0.21	38	10	0.99	32	16	0.05						
High	7	19		21	5		11	15							
															
*ALDH1*															
Low	20	23	0.09	34	9	0.99	24	19	0.81	25	18	0.21			
High	8	23		25	6		19	12		23	8				
															
*MIB-1*															
Low	17	16	0.03	30	3	0.04	20	13	0.81	26	27	0.02	19	14	0.99
High	11	30		29	12		23	18		22	19		24	17	

Abbreviation: ALDH1=aldehyde dehydrogenase isoform 1A1.

**Table 3:** Association of cancer stem cell markers with histopathological variables

	**CD44**			**CD24**			**Oct4**			**Integrin-** *β* **1**			**ALDH1**		
	**Low**	**High**	***P*-value**	**Low**	**High**	***P*-value**	**Low**	**High**	***P*-value**	**Low**	**High**	***P*-value**	**Low**	**High**	***P*-value**
*Location*															
Hypopharynx	3	3		5	1		3	3		5	1		5	1	
Larynx	11	26		27	10		25	15		24	13		19	18	
Nodal^a^	5	3	>0.23	7	1	>0.24	4	4	>0.70	4	4	>0.45	5	3	>0.20
Nasopharynx	6	2		6	2		5	3		6	2		4	4	
Oropharynx	2	11		12	1		8	5		8	5		8	5	
Parotid	1	1		2	0		1	1		1	1		2	0	
															
*T-stage*															
T1, 2	7	11		17	1		14	4		11	7		11	7	
T3, 4	16	29	0.99^b^	33	12	0.08^b^	25	20	0.15^b^	32	13	0.55^b^	25	20	0.78^b^
Unknown	5	1	0.14^c^	5	1	0.99^c^	4	2	0.01^c^	4	2	0.04^c^	4	2	0.99^c^
Recurrent	0	5		4	1		0	5		1	4		3	2	
															
*N-stage*															
N-negative	13	26	0.47	32	7	0.77	25	14	0.34	26	13	0.80	24	15	0.63
N-positive	15	20		27	8		18	17		22	13		19	16	
															
*Grade*															
1, 2	11	30	0.02	32	9	0.68	26	15	0.34	25	16	0.47	26	15	0.34
3	17	16		27	6		17	16		23	10		17	16	

Abbreviation: ALDH1=aldehyde dehydrogenase isoform 1A1.

a Unknown primary

b T1, 2 *vs* T3, 4.

c Recurrent *vs* all.

**Table 4:** Association of stem cell marker expression and of histopathological variables with response to radio-cehmotherapy (CR *vs* ICR)

	**CR**	**ICR**	***P*-value**
*T-stage*			
1,2	17	1	0.28
3,4	38	7	
			
*N-stage*			
Negative	35	4	0.04
Positive	25	10	
			
*Grade*			
1,2	32	9	0.55
3	28	5	
			
*CD44*			
Low	26	2	0.04
High	34	12	
			
*CD24*			
Low	47	12	0.72
High	13	2	
			
*Oct4*			
Low	38	5	0.07
High	22	9	
			
*Integrin-β1*			
Low	42	6	0.06
High	18	8	
			
*ALDH1*			
Low	34	9	0.76
High	26	5	
			
*MIB-1*			
Low	27	6	0.99
High	33	8	

Abbreviations: ALDH1=aldehyde dehydrogenase isoform 1A1; CR=complete response; ICR=incomplete response.

**Table 5:** Univariate and multivariate analysis of local relapse-free, overall disease-specific survival and of metastasis-free survival

	**Local relapse-free survival**		**Metastasis free-survival**		**Overall survival**	
	**Risk-ratio**	***P*-value**	**Risk-ratio**	***P*-value**	**Risk-ratio**	***P*-value**
*Univariate (Kaplan–Meier) analysis*						
Parameter						
T-stage	2.85	0.02	1.12	0.89	2.12	0.06
N-stage	2.38	0.02	3.20	0.05	1.66	0.16
Grade	1.10	0.81	1.56	0.47	1.12	0.75
CD44	2.17	0.02	0.57	0.36	1.51	0.24
CD24	1.33	0.52	0.76	0.87	1.18	0.70
Oct4	2.85	0.005	3.20	0.05	1.88	0.08
Integrin-*β*1	4.54	0.0002	8.30	0.001	2.56	0.01
ALDH1	0.43	0.02	0.43	0.17	0.55	0.09
MIB-1	1.29	0.50	1.50	0.50	0.90	0.77
						
*Multivariate (Cox regression) analysis*						
Parameter						
T-stage	5.19	0.009				
N-stage	2.03	0.08	2.68	0.14		
Grade						
CD44	1.91	0.22				
CD24						
Oct4	1.05	0.89	2.38	0.17		
Integrin-*β*1	3.08	0.007	5.72	0.01		
ALDH1	0.49	0.01				
MIB-1						

Abbreviation: ALDH1=aldehyde dehydrogenase isoform 1A1.

Results refer to comparison of high *vs* low groups. Multivariate analysis included only parameters significant at univariate.
